# Pharmacological options to relieve congestion in acute heart failure

**DOI:** 10.1007/s10741-025-10548-5

**Published:** 2025-08-30

**Authors:** Chris J. Kapelios, Ali Vazir, Lars H. Lund, Gerasimos Filippatos, James C. Fang

**Affiliations:** 1 Heart Failure Unit, Department of Cardiology, University Hospital Attikon, National and Kapodistrian University of Athens School of Medicine, Rimini 1, 12462 Haidari, Athens, Greece; 2 Heart Failure & Transplantation Unit, Onassis Cardiac Surgery Center, Athens, Greece; 3 Department of Cardiology, Royal Brompton and Harefield Hospitals, Part of Guy’s and St Thomas’ NHS Foundation Trust, London, United Kingdom; 4 National Heart and Lung Institute, Imperial College London, London, United Kingdom; 5 Unit of Cardiology, Department of Medicine, Karolinska Institutet, and Heart and Vascular Theme, Karolinska University Hospital, Stockholm, Sweden; 6 University of Utah Health Sciences Center, Salt Lake City, UT USA

**Keywords:** Diuretics, Decongestion, Acute heart failure, Volume overload, Outcomes

## Abstract

Although congestion is present in the large majority of patients hospitalized with acute heart failure (AHF), the pharmacological options to treat it remain poorly studied, with heterogeneity in real-world practices and outcomes. The best available evidence supports that patients with AHF and congestion should be initially treated with i.v. loop diuretics with their dose tailored to early (within 2–6 h) diuretic response, as assessed by spot urine sodium and/or hourly urine output. If diuretic response is sub-optimal, the next best steps seem to be increases in i.v. loop diuretics and addition of a thiazide and/or i.v. acetazolamide. Irrespective of the above, sodium-glucose co-transporter-2 inhibitors and spironolactone should be started in all patients with AHF as early as possible. Changes in serum creatinine in this scenario do not typically represent true worsening in renal function and should, thus, not lead to de-escalation of decongestion therapy.

## Introduction

Acute heart failure (AHF) carries a high burden of morbidity and mortality [1, 2], typically requiring hospital admission and representing the main driver of healthcare costs for patients with heart failure (HF) [3].

Phenotypes of AHF are heterogenous but nearly 90% of hospitalized patients present with symptoms and/or signs of congestion [4]. Although reliable assessment of volume status in patients with HF is challenging, and a multi-parametric approach is proposed [5], more than one third of the patients are discharged from the hospital with residual symptoms/signs of congestion [4], a condition associated with higher re-admission and mortality rates [4, 6]. Thus, prompt and complete relief of congestion has been identified as an important therapeutic target for these patients, aiming at improving symptoms, optimizing outcomes and reducing associated healthcare costs. However, contemporary real-world data demonstrate that there is substantial variability in decongestion strategies of patients with AHF [7, 8], likely attributable to the lack of robust evidence to guide treatment decisions.

The rationale of this review is to approach the existing evidence for pharmacological, diuretic- and non-diuretic-based, decongestion strategies in AHF. The focus of this review lies solely on the in-hospital, pharmacological, decongestive management of AHF; discussion of non-pharmacological measures or diuretics’ use in the setting of chronic HF is intentionally cursory and the reader is referred elsewhere for relevant reviews [9, 10].

## Diuretics

**Fig. 1:**
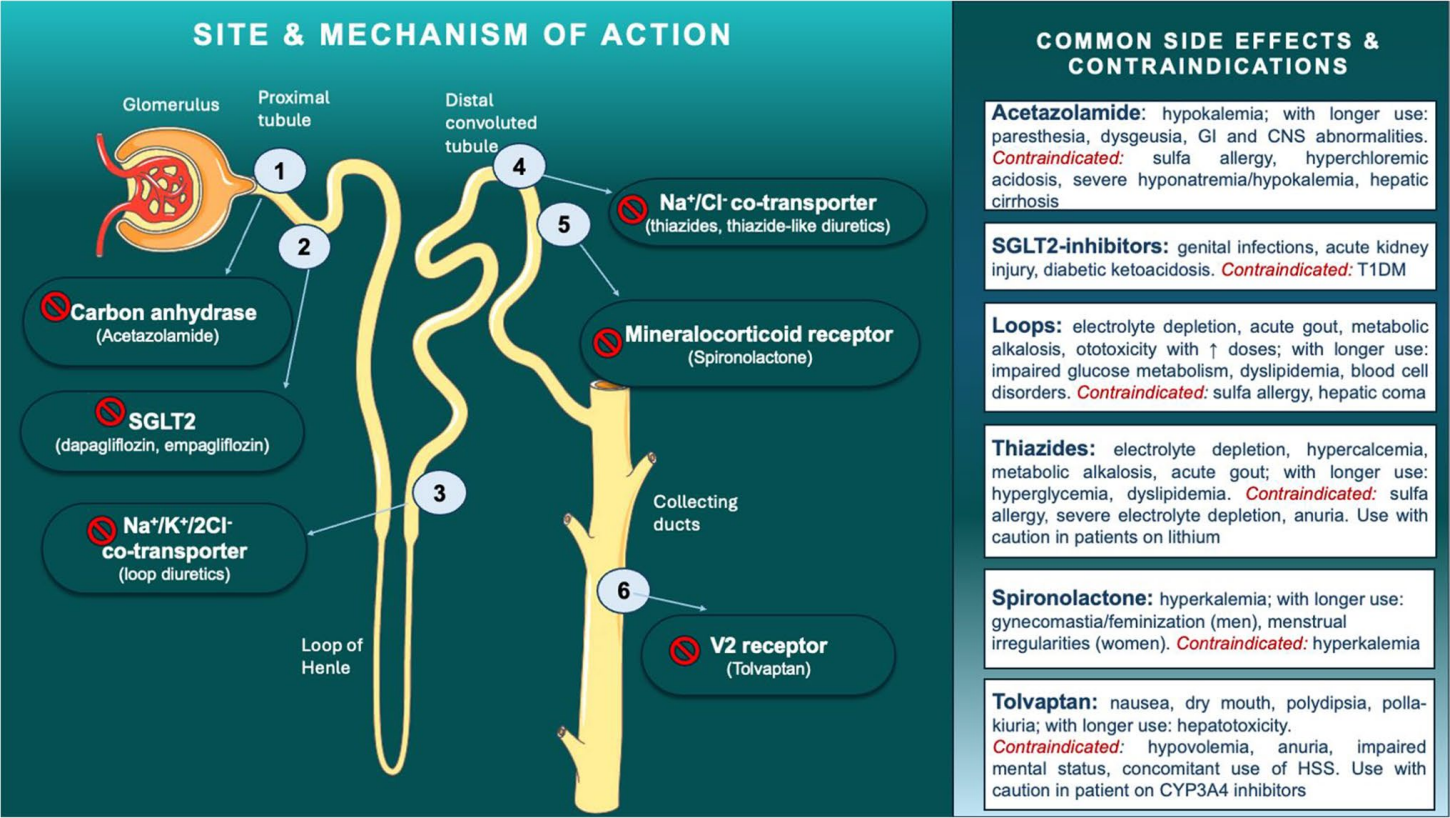
Type, mechanism of action, common side effects and contraindications of the most relevant to acute heart failure diuretics

Diuretics have different potency of natriuresis and decongestion, based on the site of the nephron where they act. The type, mechanism of action, common side effects and contraindications of the most relevant to HF diuretics are summarized in Fig. 1.

## A. Head-to-head comparison of diuretics

There is paucity of head-to-head studies of loop diuretics vs other decongestive agent monotherapy in the setting of AHF. Nonetheless, based on the cumulative clinical experience, the extrapolation of results of small studies in patients with chronic HF, and the higher likelihood of thiazides to cause electrolyte abnormalities [11], i.v. loop diuretics are recommended as first line agents for decongestion of patients with AHF [12, 13].

## B. Placebo-controlled trials of loop diuretics

There are no randomized studies of diuretics vs placebo in the setting of AHF. However, the clinical experience that diuretics are highly effective in providing prompt relief of symptoms and signs of volume overload is well established and longstanding. The literature demonstrating the efficacy of diuretics even in chronic HF is limited and result extrapolation is hindered by methodological issues [14, 15].

## C. Dosing of loop diuretics

The Diuretic Optimization Strategies Evaluation (DOSE) trial is the largest randomized controlled trial (RCT) to evaluate initial diuretic strategies in patients with acutely decompensated HF (ADHF) (Table 1) [16]. The study found no significant difference in either global assessment of symptoms or change in serum creatinine over 72 h (co-primary endpoints), with bolus vs continuous furosemide administration or with high vs low furosemide dose. However, patients randomly assigned to the high dose had more effective decongestion (dyspnea improvement, body weight loss, net fluid loss) at 72 h. Although patients in this arm more frequently presented with increases in serum creatinine by > 0.3 mg/dL within 72 h (23% versus 14%, *p* = 0.04), our current understanding is that such increases typically reflect transient, if any, worsening of renal function (WRF) [17–20], and should not be used as clinically relevant endpoints or deter decongestion [21, 22]. It needs to be highlighted that patients randomized to boluses received higher cumulative doses of furosemide (518 [292, 832] mg vs 406 [240, 628] mg, *p* = 0.008) and more frequently received thiazides (16% vs 7%, *p* = 0.02) compared with patients on infusions, implying lower diuretic efficiency with boluses [16, 23]. The trial data suggest that using higher doses of i.v. diuretics on admission (2.5 × vs 1 × oral home dose) leads to higher rates of early decongestion and should be favored, whereas a strategy of bolus vs continuous furosemide infusion is equivalent, with the former strategy likely needing more frequent treatment intensification to achieve the same effect. Therefore, when a high dose of diuretics is intended, it might be prudent to opt for an infusion or more frequent (up to q.i.d) boluses. A significant proportion of patients admitted for AHF are not treated with a loop diuretic prior to hospitalization [24, 25]. For this subset of patients the expert consensus is for initial dosing of an i.v. loop diuretic bolus equal to 20–40 mg of furosemide [26]. However, recent data demonstrated that diuretic response was better when higher, natriuretic-guided doses of loop diuretics were used, both among patients previously receiving and those naïve to diuretics [24], hinting that a more aggressive initial approach may be warranted, especially when assessment of diuretic response is expected to be delayed, as often happens in clinical practice [27]. Interestingly, the median dose of i.v. loop diuretics over the first 24 h in the intervention arm of the Pragmatic Urinary Sodium-based algoritHm in Acute Heart Failure (PUSH-AHF) trial was equal to 6 times the median oral dose of patients pre-treated with diuretics [24]. Importantly, this practice appears safe without issues of hypotension or declines in renal function.

**Table 1:** Main characteristics of selected decongestion studies

Study (refe-rence)	N	Design	Patient popular-tion	Duration of interven-tion	Endpoint assessment timepoints	Primary endpoint	Baseline chara-cteristics	Main efficacy outcomes	Main safety outcomes
DOSE (16)	308	−2 × 2 factorial design -i.v. furosemide as a) continuous infusion vs BID boluses and b) high (2·5 × PO home dose) or low dose (1 × PO home dose)	Inclusion: -Admitted with ADHF within last 24 h -Hx of CHF -PO LD at daily dose of 80–240 mg in FE -Thiazides permitted if taken long-term Exclusion: -SBP < 90 mm Hg -sCr > 3 mg/dl -i.v. vasodilators or inotropes	−72 h -prespecified dose changes permitted after 48 h	−72 h for decongestion outcomes −60d for hard outcomes	1) VAS of global assessment of symptoms 2) change in sCr from baseline to 72 h	-Age 66y -Female 27% -NYHA nr -LVEF 35% -NTproBNP 7433 pg/ml eGFR nr	-No difference in VAS -No difference in hard outcomes (LOS and composite of death, HFH or ED visit) -Greater relief of dyspnea (p = 0·04), ↓ in BW (p = 0·01), net fluid loss (p = 0·001) and a trend towards a greater ↓ in NT-proBNP (p = 0·06) with high dose	WRF (↑sCR > 0·3 mg/dl) in 23% with high dose vs 14% with low dose (p = 0·04)
CLOROTIC (31)	230	−1:1 to PO HCTZ at a stable dose adjusted to eGFR (25 mg for eGFR > 50 ml/min; 50 mg for eGFR 20–50 ml/min; 100 mg for eGFR < 20 ml/min) or placebo QD -Background i.v. furosemide 1 × PO home dose divided in BID boluses	Inclusion: -Admitted with ADHF within last 24 h -Hx of CHF - PO LD at daily dose of 80–240 mg in FE Exclusion: -Instability on admission -i.v. inotropes -Thiazide within 1 m -RRT -K ≤ 2·5 mmoL/L -Na ≤ 125 mmoL/L	−5 days -No change to HCTZ unless driven by eGFR changes -Prespecified dose changes to loops permitted after at 24 and 48 h -Changes to loops at physician discretion after 72 h	−72 and 96 h for decongestion outcomes −30d and 90 d for hard outcomes	1) changes in BW from baseline to 72 h 2) changes in patient-reported dyspnea from baseline to 72 h	Age 83 y Female 48% NYHA II/III 86% LVEF 56%* NT-proBNP 4525*pg/ml eGFR 43 ml/min/1·73m^2^*	Greater ↓ in BW with HCTZ (−2·3 vs −1·5 kg, p = 0·002) Greater diuresis (1·77 vs 1·4 L, p = 0·05) -No difference in AUC for dyspnea -No difference in hard outcomes (death, HFH)	Hypokalemia (K ≤ 3·5 mmol/L) in 44·7 vs 19%, (p < 0·001) -WRF (↑sCr > 0·3 mg/dl) in 46·5 vs 17·2% (p < 0·001) but not difference in ↓ eGFR > 50% (p = 1·000)
ADVOR (41)	519	−1:1 to i.v. ACZ (500 mg QD) or placebo - Background i.v. loops 2 × PO home dose, as a single bolus immediately after randomization and split into BID boluses thereafter (max permitted bolus dose 100 mg FE)	Inclusion: -Admitted with ADHF + ≥ 1 sign of volume overload + NT-proBNP ≥ 1000 pg/ml or BNP ≥ 250 pg/ml - PO LD at daily dose of ≥ 40 mg in FE Exclusion: -SBP < 90 mmHg -prior receipt of ACTZ or SGLT2i -eGFR < 20 ml/min/1·73m^2^ -IV LD > 80 mg prior to randomization	- Up to 72 h - If UO after 30-48 h < 3·5 L + signs of volume overload escalation of decongestion was mandatory (physician could select from 3 pre-defined options)	−72 h for decongestion outcomes −90d for hard outcomes	Successful decongestion (defined as absence of signs of volume overload assessed with generic congestion score) within 3 days after randomization without an indication for escalation of decongestion	Age 78 y Female 37% NYHA II/III 70% LVEF 43% NT-proBNP 6173 pg/ml eGFR 39 ml/min/1·73m^2^*	-Successful decongestion within 72 h (RR: 1·46; p < 0·001) and more likely with ACTZ - ↑ diuresis and natriuresis within 48 h - ↓ LOS (8·8 vs 9·9d) - No difference in mortality or HFHs	-Hypokalemia (K^+^ < 3 mmol/L) at 90 d 23·4% with ACTZ vs 14·7% with placebo (p = 0·0134) -No difference in other safety endpoints
ATHENA-HF (48)	360	1:1 to PO spironolactone 100 mg QD vs placebo (for pts naïve to spironolactone) or spironolactone 25 mg QD (for pts previously receiving spironolactone) - Background i.v. loop Tx left at physician’s discretion	Inclusion: -Clinical diagnosis of HF + 1 symptom + 1 sign of ADHF + NT-proBNP ≥ 1000 pg/ml or BNP ≥ 250 pg/ml within 24 h of randomization Exclusion: -On Tx with eplerenone - On Tx with spironolactone at daily dose > 25 mg -SBP ≤ 90 mmHg -serum K^+^ > 5 mmol/L -eGFR < 30 ml/min/1·73m^2^	−96 h -Study drug discontinued after 96 h and further MRA use left to physician’s discretion	-Up to 96 h for decongestion outcomes −30d for hard outcomes	Proportional change in the log NT-proBNP from randomization to 96 h (or to hospital discharge, whichever came first)	Age 65 y Female 36% NYHA II/III nr LVEF 33%* NT-proBNP 4102 pg/ml* eGFR 57 ml/min/1·73m^2^*	- No difference in log NT-proBNP or other decongestion endpoints (dyspnea, congestion score, net UO, BW change, need for loops, WHF) -No difference in time to death, HFH or urgent ED visit	- No difference in WRF (↑sCr > 0·3 mg/dl), hyperkalemia, or SAEs
DICTATE-AHF (53)	240	1:1 to PO dapagliflozin 10 mg QD or structured usual care - If pts not on loops prior to randomization initial daily i.v. loops dose 2–2·5 × PO home dose divided into BID administration Thereafter dose was adjusted every 12–24 h based on titration protocol with UO target of 3–5 L/d in both arms Daily i.v. loops increased to ≥ 960 mg in FEs before a thiazide was added - Use of MRA at dose ≥ 100 mg/d, non-study thiazide or ACTZ was not permitted	Inclusion: -Admitted with AHF within last 24 h - ≥ 2 objective measures of hypervolemia - T2DM (not obligatory after protocol amendment) -eGFR ≥ 30 mL/min/1·73 m^2^ (≥ 25 mL/min/1·73 m^2^ with protocol amendment) -current or planned Tx with i.v. loops Exclusion: -T1DM -SBP < 90 mm Hg -sGlu < 80 mg/dL -Hx of diabetic ketoacidosis	D5 or discharge, whichever came first	- D5 or discharge, whichever came first, for decongestion outcomes −30d for hard outcomes	- DE (cumulative change in BW/cumulative i.v. + PO loop dose) from enrollment to D5 or discharge, whichever came first	Age 65 y* Female 39% NYHA II/III nr LVEF 40%* NT-proBNP 2602 pg/ml* eGFR 53 ml/min/1·73m^2^*	- No difference in OR for improved DE between dapagliflozin and usual care (OR: 0·65; 95% CI: 0·41–1·02; p = 0·06) -No difference in BW change (p = 1·00) but ↓ cumulative LD dose with dapagliflozin (median 560 [260,1150] mg FEs vs 800 [380,1715] mg, p = 0·006) - No difference in clinical outcomes (in-hospital WHF, AHF at 30 d)	- No difference in change in eGFR at end of study (−2 ml/min/1·73m^2^ with dapagliflozin vs −3·2 ml/min/1·73m^2^ with usual care, p = 0·79)

* Values are means of the 2 group median values

†ACS: acute coronary syndrome; ACZ: acetazolamide; ADHF: acute decompensated heart failure; AE: adverse event; AHF: acute heart failure; AUC: area under the curve; BID: twice daily; CHF: chronic heart failure; DE: diuretic efficiency; ED: emergency department; eGFR: estimated glomerular filtration rate; FE: furosemide equivalents; HF: heart failure; HFH: heart failure hospitalization; Hx: history; i.v.: intravenous; LD: loop diuretic; LVEF: left ventricular ejection fraction; MRA: mineralocorticoid receptor antagonist; NYHA: New York Heart Association; OR: odds ratio; PO: oral; QD: once daily; RRT: renal replacement therapy; SAE: serious adverse event; SBP: systolic blood pressure; sCr: serum creatinine; SGLT: sodium glucose transporter; sGlu: serum glucose; T1DM: type 1 diabetes mellitus; T2DM: type 2 diabetes mellitus; Tx: treatment; UNa: urine sodium; UO: urine output; VAS: visual analog scale; WHF: worsening heart failure; WRF: worsening renal function

## D. Type of loop diuretic

Although previously suggested that pharmacological differences of loop diuretics may translate into differences in outcomes, the recently published TRANSFORM-HF (Torsemide Comparison With Furosemide for Management of Heart Failure) trial demonstrated that in patients discharged after a HF admission, torsemide compared with furosemide did not result in a difference in all-cause mortality or any other study endpoint over 12 months [28]. When focusing on the in-hospital setting, no data from RCTs are available. Interestingly though, while the diuretic dose equivalence of furosemide and bumetanide has been long defined, selection of bumetanide as the initial i.v. loop diuretic seems to be associated with relative underdosing of the medication [29]. In light of the lack of data from RCTs, furosemide, torsemide, and bumetanide can be used interchangeably, as long as the correct dose conversion equation. (80 mg oral furosemide = 40 mg i.v. furosemide = 20 mg torsemide oral or i.v. = 1 mg bumetanide oral or i.v.) is utilized. Importantly, a recent mechanistic study demonstrated that the diuretic equipotent dose ratio between oral furosemide and oral torsemide is 4:1 and not 2:1 as commonly used in clinical practice [30].

## Combination of loop diuretic with other diuretic agents

### A. Loop diuretics and thiazides/thiazide-like diuretics

The Combination of Loop with Thiazide-type Diuretics in Patients with Decompensated Heart Failure (CLOROTIC) trial randomly assigned patients hospitalized for ADHF previously on oral loop diuretics to oral hydrochlorothiazide (HCTZ) at a dose adjusted to their estimated glomerular filtration rate (eGFR) or placebo once daily for 5 days (Table 1) [31]. All patients received i.v. loop diuretics at a dosing scheme resembling the low-dose arm of DOSE trial. The study demonstrated a greater loss of body weight with HCTZ but no difference in the co-primary endpoint of AUC for dyspnea at 72 h compared with placebo [31]. The use of HCTZ was associated with improved metrics of diuretic response, but no difference in hard clinical outcomes. HCTZ use caused significantly more hypokalemia and increases in serum creatinine > 0.3 mg/dl but not decreases in eGFR > 50% compared with placebo. The trial data suggest that addition of HCTZ to a low-dose i.v. loop diuretic regimen is relatively safe and can lead to greater early weight loss without affecting length of stay or post-discharge outcomes. The decision of the investigators to compare the combination of HCTZ with a fixed, low dose of loop diuretics rather than with the recommended standard of care (rapid, escalation of loop diuretic dose if diuresis/natriuresis is insufficient) [12, 26], does not permit defining the exact place that HCTZ should have in the optimal decongestion algorithm. However, based on the relative safety of higher doses of loop diuretics and the tendency of thiazides to cause electrolyte disturbances,(31,32) it seems reasonable to prioritize loop diuretic dose increase over addition of thiazides in the decongestion of patients with ADHF on low-dose loop diuretics, particularly when furosemide dosing is < 80 mg daily [26].

The recently published DEA-HF randomized study compared 3 different diuretic regimens in patients with worsening HF (WHF) necessitating intermittent, i.v. diuretics in the ambulatory setting [32]. The study showed a superior diuretic effect in terms of natriuresis and weight loss with furosemide + metolazone relative to furosemide monotherapy [32]. The two interventions were equally safe pertaining to the incidence of electrolyte disturbances, hypotension and acidosis. However, serum creatinine increases were more often in the combination compared with the monotherapy arm, a finding with uncertain clinical implications, as previously discussed [32].

Chlorothalidone and metolazone are two thiazide-type drugs that block the sodium-chloride co-transporter in the distal convoluted tubule. Available data suggest similar decongestive effects for metolazone and i.v. chlorothiazide (CTZ) (dose ratio CTZ 1000–2000 mg: metolazone 10 mg) [33–35]. Thus, these two agents can be used interchangeably, although metolazone is substantially less costly [36]. Although direct comparisons of HCTZ with metolazone are lacking, a small RCT compared the effect of metolazone vs bendroflumethiazide (10 mg PO daily for both), in patients with NYHA III/IV HF who failed to respond to ≥ 80 mg i.v. furosemide b.i.d. Both agents resulted in similar decongestion as assessed by decreases in body weight [37]. In light of the equipotency of appropriately dosed thiazide/thiazide-like diuretics [38], it seems reasonable to prioritize PO HCTZ or metolazone over i.v. CTZ, in patients who do not respond to adequate doses of loop diuretics.

### B. Loop diuretics and acetazolamide

Acetazolamide (ACZ) is generally a weak diuretic that inhibits proximal sodium reabsorption [39]. However, some studies demonstrated that ACZ modestly augments the natriuretic/diuretic effect of loop diuretics [40]. The use of ACZ in this manner was explored in the Acetazolamide in Acute Decompensated Heart Failure with Volume Overload (ADVOR) trial which randomly assigned patients with ADHF to receive i.v. either ACZ (500 mg once daily) or placebo on top of standardized i.v. loop diuretics (Table 1) [41]. The trial demonstrated that ACZ use resulted in 46% increased likelihood of achieving decongestion over the 72-h treatment period compared with placebo (primary endpoint). This effect was consistent among prespecified subgroups except those with pre-admission oral loop diuretic dose > 60 mg furosemide equivalents (FEs) daily, a finding with potential clinical relevance. Cumulative diuresis and natriuresis were also significantly higher in the ACZ group. There was no significant difference in rates of adverse events between the groups although 90-day hypokalemia occurred more often in the ACZ arm. All-cause mortality and HF hospitalization rates did not differ significantly between study groups, although there was a nominally higher rate of death at 3 months with ACZ (15.2% vs 12.0%). However, the generalizability of these results has been challenged [42, 43]. Furthermore, DEA-HF showed a superior natriuretic and diuretic effect of furosemide + metolazone relative to furosemide + ACZ in ambulatory patients with WHF [32]. In patients with elevated plasma HCO_3-_ (e.g., ≥ 27 mmol/l), ACZ could be prioritized, as a pre-specified sub-analysis of ADVOR suggested that benefit is more pronounced in those with plasma HCO_3-_ ≥ 27 mmol/l [44]. The most recent update of guideline recommendations did not specifically address how to use ACZ in AHF, underlining the need for more data [45].

### C. Loop diuretics and spironolactone

Spironolactone’s use in HF dates back to the 1990s. Spironolactone in doses 50–200 mg daily has been safely used and associated with greater decongestion in HF patients resistant to loop diuretics ± metolazone [46, 47]. However, in the phase II, Aldosterone Targeted Neurohormonal Combined with Natriuresis Therapy in Heart Failure (ATHENA-HF) trial, spironolactone failed to meet the primary endpoint of decreasing the 96-h NT-proBNP in patients randomized to spironolactone 100 mg daily vs placebo [or low-dose (25 mg daily)], although NT-proBNP declined considerably in both groups (Table 1) [48]. Predictably, substantially less potassium supplementation (*p* < 0.001) was needed at 48, 72 and 96 h in the treatment vs the control arm [49].

Although the study’s neutral results led to spironolactone being removed from the treatment algorithm of the most recent guidelines [12], several points relative to the study design have previously raised concern [50]. In any case, ATHENA-HF demonstrated that inpatient administration of spironolactone 100 mg daily was safe, while reducing the need for potassium supplementation [49]. More recently, small studies reported that in-hospital administration of spironolactone in doses up to 300 mg daily was safe and associated with better decongestion in ADHF patients [50, 51]. Whether higher doses of spironolactone in this range can be used safely as adjunct treatment in patients with ADHF and diuretic resistance awaits further study.

In summary, spironolactone, and likely other MRAs, at a dose up to 100 mg daily can be safely used in patients with ADHF to prevent excessive hypokalemia. The optimal dose for natriuretic augmentation of standard loop diuretics awaits further study.

### D. Loop diuretics and sodium-glucose co-transporter-2 inhibitors

Natriuresis/diuresis was one of the first mechanisms postulated to underlie the beneficial effects of sodium-glucose co-transporter-2 inhibitors (SGLT-2i) in patients with HF. Although the safety and efficacy of SGLT-2i in chronic HF are well documented and reflected by the strong recommendations in the latest guidelines [12, 13, 45], data in the AHF setting were until recently sparse. EMPULSE (EMPagliflozin in patients hospitalized with acUte heart faiLure who have been StabilizEd) trial, though not a decongestion study per se, proved that initiation of empagliflozin 10 mg daily a median 3 [2–4] days following a HF admission was safe and resulted in improved decongestion through days 15, 30, and 90 [52]. Similarly, the recent RCT, DICTATE-AHF (Efficacy and Safety of Dapagliflozin in Acute Heart Failure) showed similar diuresis, natriuresis and body weight loss when comparing dapagliflozin 10 mg daily vs placebo on top of an aggressive i.v. loop diuretics ± metolazone decongestion protocol (Table 1) [53]. Higher cumulative loop diuretic doses in the placebo arm where needed, translating to improved diuretic efficiency with dapagliflozin. No difference was noted between the 2 study arms in the incidence of adverse events or hard clinical outcomes.

In another small RCT 61 patients with AHF and diuretic resistance were assigned 1:1 to 3-day treatment with either dapagliflozin 10 mg or metolazone 5–10 mg daily [54]. The study demonstrated that the two treatment approaches exerted the same decongestion effects but with higher loop diuretic efficiency in the metolazone arm. Metolazone caused more pronounced changes in levels of serum urea, creatinine, sodium and potassium but no difference in the incidence of adverse events, or clinical outcomes was reported [54]. Another interesting finding of the study was that the proportion of patients enrolled to those excluded from the study due to absence of diuretic resistance was ~ 1:10, suggesting that the large majority of AHF patients can be effectively decongested with adequate doses of i.v. loop diuretic monotherapy [54].

Based on the above, we suggest an SGLT2i be added at the earliest timepoint possible to the treatment of patients with AHF to increase diuretic efficiency, as well as to provide long-term clinical benefits of decreased HF re-hospitalizations and cardiovascular mortality.

## Combination of loop diuretic with non-diuretic agents

### Α. Loop diuretics and saline infusions

Decreased plasma chloride has been associated with poor outcomes in HF and the renal chloride sensor may play a significant role in renal sodium avidity [55]. Small studies have assessed the safety and efficacy of the combination of hypertonic saline solutions (HSS) infusion + loop diuretics vs loop diuretics ± placebo [56]. A meta-analysis of these studies showed greater weight loss with a renoprotective effect of HSS compared with control, which was also paralleled by a reduction in length of stay and improved outcomes [56]. Similar findings were noted in a single center experience of 58 ADHF patients with diuretic resistance treated with HSS (150 ml 3% NaCl over 30 min) in addition to high-dose loop diuretics ± thiazides [57]. In the recently published, multi-center, SALT-HF trial, a 1-h infusion of HSS + furosemide did not result in superior short-term decongestion or better outcomes compared with 1-h infusion of furosemide alone among 167 ambulatory patients with WHF [58]. However, greater weight loss at 7 days was noted in the intervention arm. Importantly, the median baseline values of serum sodium and chloride were within normal range. In another small RCT oral NaCl (2gr t.i.d.) did not improve decongestion vs placebo when used with i.v. loop diuretics in patients with AHF [59]. However, a recent, single-center, single-blinded trial randomly allocating 50 congested, AHF with no major electrolyte abnormalities to a 2 L daily infusion of either normal saline 0.9% or 5% glucose on top of a conservative intravenous diuretic regimen, demonstrated significantly greater decongestion with a higher diuretic efficiency in the former group [60]. The mechanisms underlying this positive effect of normal saline were suppressed sodium reabsorption in the proximal tubule without a compensatory increase in distal reabsorption, and lower concentrations of serum aldosterone.

Whether or not congested patients-hypochloremic and/or hyponatremic or not- despite high-dose loop diuretics, SGLT2i, thiazides, ACZ and spironolactone, would benefit from saline infusions remains investigational.

### B. Loop diuretics and aquaretics

Vasopressin-2 receptor antagonist prevents the resorption of free water at the renal collecting ducts. This promotes free water clearance and leads to increases in serum sodium in cases of dilutional hyponatremia. In the Efficacy of Vasopressin Antagonism in Heart Failure Outcome Study with Tolvaptan (EVEREST) trial, although tolvaptan 30 mg daily vs placebo had no effect on clinical outcomes [61], it led to superior decongestion (improved dyspnea, weight loss, relief of edema) either at day 1 or day 7 [62]. The TACTICS-HF (Targeting Acute Congestion with Tolvaptan in Congestive Heart Failure) study [63], which randomized 257 patients with ADHF within 24 h of their presentation to tolvaptan 30 mg or placebo on top of a fixed, for the first 48 h, i.v. loop diuretic regimen, showed significantly greater weight loss and diuresis with tolvaptan at 24 and 48 h, although no difference in the improvement of dyspnea, which represented the study’s primary outcomes, was noted [63]. Importantly, the background loop diuretic treatment was conservative, capping maximum daily diuretic at 80 mg FEs. In the small 3 T study, the addition of tolvaptan 30 mg daily on top of an aggressive i.v. loop diuretic regimen produced significantly less 48-h spot natriuresis but similar effects on diuresis and body weight loss compared with metolazone or CTZ [34]. A recent systematic review on diuretic potentiation strategies concluded that tolvaptan reduced body weight and improved dyspnea in the RCTs it was tested in [64].

The existing data suggest that the use of tolvaptan should be reserved for its currently approved indications, as its routine use as a decongestive agent is not supported by the existing evidence base.

## Decongestion tailoring markers

Clinical and echocardiographic parameters have been shown to be unreliable in assessing and grading congestion when used in isolation [26], thus contributing to the wide variability of their use in clinical practice [7]. Several clinical congestion scores have been developed [5], but have not been established as standard of care. Novel biomarkers such as ST2, CA-125, bio-atienomedullin, and CD-146 may be useful in quantifying congestion and differentiating types of congestion, but are not widely used [65, 66]. Several limitations also apply to the parameters used to monitor diuretic response, the most frequently used of which are rather insensitive and slowly affected (body weight), or challenging to monitor reliably (urine output [UO]) [26]. Several observational studies have associated poor natriuresis following diuretic administration with adverse clinical outcomes in patients with AHF [67, 68]. Moreover, a net negative sodium, but not fluid, balance has been correlated with clinical outcomes [67]. Therefore, urine sodium has been proposed both as a surrogate marker of diuretic response in clinical practice [12, 26], and a relevant endpoint in recent decongestion trials [24, 41, 53]. Natriuresis-guided decongestion was demonstrated to lead to superior short-term natriuresis and diuresis compared with standard or care [24, 69], while also resulting in shorter length of stay when used as part of a standardized decongestion protocol [69]. However, no difference in hard clinical outcomes was shown. The EASY-HF study demonstrated that a nurse-led, natriuresis-guided decongestion protocol with use of a point-of-care urinary sodium sensor was feasible, safe and led to better decongestion in terms of natriuresis and diuresis at 48-h compared to physician-led standard of care [70]. The introduction of point-of-care urinary sodium measurements may help tackle the logistical constraints hindering the use of natriuresis-guided decongestion in every-day practice [7, 70]. Importantly, the studies performed to date have not provided the evidence base to support that natriuresis-guided decongestion leads to improved clinical outcomes. Therefore, traditional markers of decongestion, such as UO, can also be used to tailor treatment, as long as they are assessed early after each intervention [12, 26].

Finally, the definition of adequate decongestion or euvolemia remains highly variable in clinical practice as well as RCTs [5]. No single metric-clinical, laboratory or imaging-performs reasonably well in isolation. In fact, inadequate decongestion despite net negative fluid loss and decreases in weight is common and likely contributes to the high rate of 30-day and 6-month readmission for congestion in HF. Therefore, the current trend is that decongestive measures should continue until symptoms and signs of volume overload resolve and multiparametric congestion assessment ($\ge$ 2 of changes in NT-proBNP, laboratory markers of hemoconcentration, cardiac echo, lung ultrasound and venous excess ultrasound) indicates euvolemia [5, 71–73]. Invasive hemodynamic assessment of filling pressures and implantable hemodynamic monitors may help to address this issue in selected patients.

## Confounding role of eGFR and creatinine during diuresis in AHF

As mentioned, WRF, as typically assessed by increases in serum creatinine, has been commonly used in AHF studies as a safety endpoint, but definitions vary [22]. In most contemporary studies, the definition for WRF of an increase in sCr > 0.3 mg/dL (> 26.5 umol/L) or/and a fall in eGFR of > 20% is typically used. However, and importantly, the use of serum creatinine is only accurate as a measure of GFR in the steady state, which is rarely true in patients admitted with AHF [74]. Moreover, acute declines in eGFR generally represent declines in glomerular pressure mediated by hemodynamic changes in afferent and efferent arteriolar tone. Thus, these acute declines in eGFR with diuretic management likely represent “pseudo-WRF” or “permissive hypercreatinemia,” which is hemodynamically transient, not harmful, and instead associated with successful decongestion in the clinical scenario of congestive AHF [22, 74]. Although often characterized as “acute kidney injury” (AKI), evidence to date suggests there is no identifiable injury to the kidney [75]. Importantly, these acute increases in sCr are not associated with poor outcomes in patients with AHF, as long as decongestion is accomplished [76] Contrary, when WRF is associated with inadequate, persistent or worsening congestion, it is associated with worse outcomes in patients admitted with AHF [77]. Ironically, transient improvements in renal function during decongestion in the AHF setting have also been associated with worse outcomes, likely reflecting the presence of greater underlying cardiorenal dysfunction and more severe congestion in this patient subset [19].

In conclusion, physicians should not de-escalate decongestive measures solely because of increases in serum creatinine, given that diuretic response is satisfactory. In contrast, when such increases parallel inadequate diuretic response, following a stepwise, diagnostic and therapeutic algorithm, which sequentially addresses, among others, reversible causes of renal insult (antimicrobials, iodized contrast etc.), inadequate decongestive measures and low-output state, is warranted [78]. In such a scenario, consultation with a HF specialist and invasive hemodynamic assessment may be considered.

## Practical guide to decongestion

**Fig. 2:**
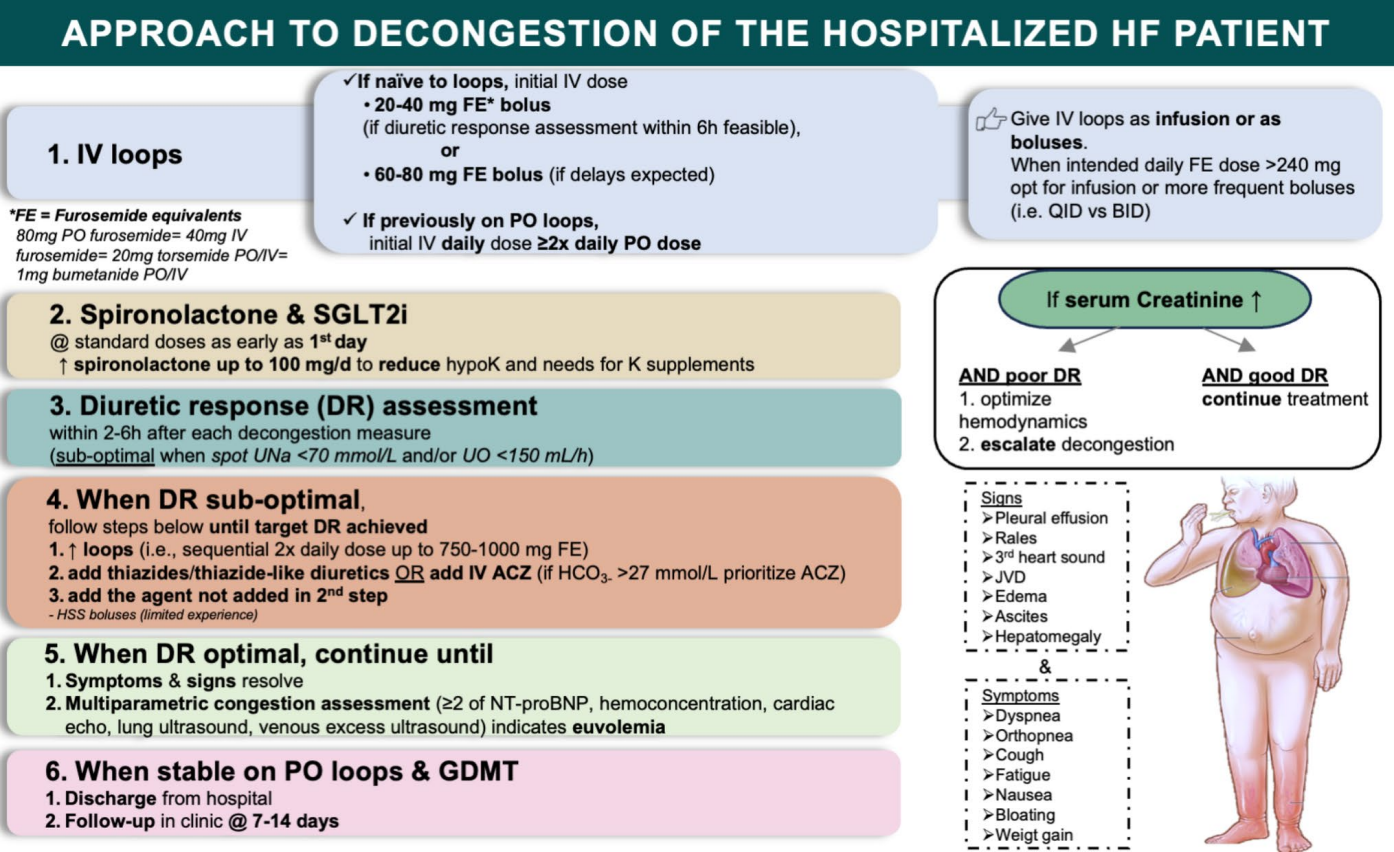
A roadmap to pharmacological decongestion in acute heart failure based on contemporary clinical data

In a patient admitted to the hospital with AHF and symptoms (e.g., dyspnea, orthopnea, weight gain) and/or signs (e.g., pleural effusion, rales, pedal edema) of congestion first choice for pharmacological decongestion should be an i.v. loop diuretic. Any loop diuretic available in i.v. formulary can be used given that the correct conversion formula is used. In diuretic-naïve patients the initial bolus dose should be 20–40 mg of FEs (or more if delays in response assessment are expected), whereas in patients on PO home loop diuretics the initial i.v. dose should be at least 2 × their daily PO dose. An SGLT2i and spironolactone should be added to the drug regimen of all patients with AHF and congestion as early as their first in-hospital day. Diuretic response (spot urine sodium and/or UO) should be assessed within 2–6 h of each new decongestive measure and when sub-optimal (spot urine sodium < 70 mmol/L and/or UO < 150 mL/h), decongestion should be further escalated. In such a scenario sequential doubling of loop diuretics (up to a maximum of 1000 mg FEs daily), followed by addition of a thiazide/thiazide-like diuretic or i.v. ACZ are the next reasonable steps. Once target diuretic response is achieved, the decongestive regimen should remain unchanged until symptoms/signs have completely resolved and multiparametric congestion assessment indicates euvolemia. At that point the patient should be transitioned to PO loop diuretics and all guideline-directed medical therapy should be added or up-titrated. Once stable, the patient should be discharged from the hospital with a follow-up appointment in HF clinic at 7–14 days with a plan to aggressively optimize GDMT, a strategy shown to promote sustainable decongestion [79]. A graphical guide to decongestion is depicted in Fig. 2.

## Summary and future directions

Although congestion represents the hallmark feature of AHF in most cases, pharmacological options to treat congestion remain poorly studied. Unfortunately, the heterogeneity seen in clinical practice is also noted within RCTs, with variable patient populations enrolled, and efficacy and safety endpoints being used, rendering the comparison of results within or across studies challenging (Table 2) [22, 64]. 

**Table 2:** Main characteristics of control arms of short-term acute heart failure trials

	DOSE	CLOROTIC	ADVOR	ATHENA-HF	EMPULSE	DICTATE-AHF
Baseline						
Age, years	66	82	79	65	70	64
Female Sex, %	27	57	40	36	35	44
NYHA III/IV, %	-	65	87	86	-	-
LVEF, %	33	57	43	30	32	35
eGFR, ml/min/1.73m^2^	-	44	38	55	54	54
NT-proBNP, pg/ml	8125	4330	6483	4176	3106	2927
After randomization						
Daily i.v. loop diuretic dose, (FE, mg)	80	80	120	160	72	240
BW loss, kg (FU in days)	2.8 (3)	1.5 (3)	-	2.8 (4)	1.2 (15)	-
Diuresis, L (FU in days)	Net 3.6 (3)	1.4 (1)	4.1 (3)	3.8 (3)	-	2.9 (1)
Successful decongestion, %	11	-	33	-	-	-
Worsening renal function^*^, %	14	17	0.8^†^	32	0.8^‡^	0.8^§^
Hypokalemia, %	-	16	3.9	-	-	0.8
HF (re)admission, % (FU in days)	36 (60)	-	17 (90)	-	4.5 (30)	6.9 (30)
Death, % (FU in days)	11 (60)	16 (90)	12 (90)	-	8.3 (90)	4.2 (30)

* Increase in sCr > 0.3 mg/dl. † Doubling of baseline sCr, ≥ 50% sustained decrease in eGFR or renal replacement therapy during index hospitalziation. ‡ Occurrence of chronic dialysis or renal transplant or sustained reduction of ≥ 40% eGFRCKD-EPIcr, or sustained eGFRCKD-EPIcr < 15 ml/min/1.73 m^2^ for patients with baseline eGFR ≥ 30 < 15 ml/min/1.73 m^2^, sustained eGFRCKD-EPIcr < 10 < 15 ml/min/1.73 m^2^ for patients with baseline eGFR < 30 ml < 15 ml/min/1.73 m^2^. § Scr increase $\geq$ 300% or renal replacement therapy. || BW: body weight; eFGR: estimated glomerular filtration rate; FE: furosemide equivalents; HF: heart failure; i.v.: intravenous; LVEF: left ventricular ejection fraction; NYHA: New York Heart Association

In line with the above, many important questions regarding the use of decongestion therapies remain unanswered (Table 3).

**Table 3:** Unanswered questions concerning decongestion in acute heart failure

1. What is the optimal initial dose of loop diuretics in patients with acute heart failure who are diuretic naïve?
2. What is the optimal loop diuretics dosing up- and down-titration algorithm?
3. What is the best next step in treatment escalation when the response to the initial loop diuretics dosing regimen is sub-optimal?
4. Which is the best combination of markers to assess congestion and tailor treatment?
5. Does decongestion based on any marker/combination of markers lead to improved clinical outcomes?
6. Does any decongestion option/measure and/or use of a decongestion protocol lead to better clinical outcomes?
7. Which is the best combination of markers to define euvolemia and permit hospital discharge?
8. What is the optimal loop diuretic dose at discharge?
9. What is the best approach to assess renal function in acute decompensated HF?
10. Can intrinsic renal disease be distinguished from HF-related renal dysfunction?

Several small studies, investigating the decongestive effect of novel approaches, such as oral steroids [80], the utility of standardized decongestive protocols [81], or the comparative efficacy of 2^nd ^line decongestive agents, such as metolazone vs ACZ (Optimal Diuretic Therapies for Acute Heart Failure With Volume Overload [DRAIN-AHF], NCT06166654), have recently been published or are currently underway. In anticipation of such studies that will enable better, evidence-based use of decongestive treatment of AHF in the future, use of a practical algorithm of pharmacological decongestion, based on the critical synthesis of available data, seems prudent (Fig. 2).

## Data Availability

No datasets were generated or analyzed during the current study.
